# Characterization of Virulence Factors of *Staphylococcus aureus*: Novel Function of Known Virulence Factors That Are Implicated in Activation of Airway Epithelial Proinflammatory Response

**DOI:** 10.4061/2011/601905

**Published:** 2011-09-14

**Authors:** Justyna Bien, Olga Sokolova, Przemyslaw Bozko

**Affiliations:** ^1^Witold Stefanski Institute of Parasitology of the Polish Academy of Sciences, Twarda Street 51/55, 00-818 Warsaw, Poland; ^2^Institute of Experimental Internal Medicine, Medical Faculty, Otto-von-Guericke University, Leipziger Str*β* 44, 39120 Magdeburg, Germany; ^3^Institute for Molecular Biology, Hannover Medical School, Carl Neuberg Str*β* 1, 30625 Hannover, Germany

## Abstract

Airway epithelial cells play a major role in initiating inflammation in response to bacterial pathogens. *S. aureus* is an important pathogen associated with activation of diverse types of infection characterized by inflammation dominated by polymorphonuclear leukocytes. This bacterium frequently causes lung infection, which is attributed to virulence factors. Many of virulence determinants associated with *S. aureus*-mediated lung infection have been known for several years. In this paper, we discuss recent advances in our understanding of known virulence factors implicated in pneumonia. We anticipate that better understanding of novel functions of known virulence factors could open the way to regulate inflammatory reactions of the epithelium and to develop effective strategies to treat *S. aureus*-induced airway diseases.

## 1. Introduction

Although a relatively unspectacular, nonmotile coccoid bacterium, *Staphylococcus aureus* is a dangerous human pathogen in both community-acquired and nosocomial infections. A fundamental biological property of this bacterium is its ability to asymptomatically colonize healthy individuals. *S. aureus *carriers are at higher risk of infection, and they are presumed to be an important source of the *S. aureus *strains that spread among individuals [1]. 

The pathogen can cause a wide variety of infections, which can be divided into three types: (i) superficial lesions such as wound infection, (ii) toxinoses such as food poisoning, scalded skin syndrome and toxic shock syndrome, and (iii) systemic and life-threatening conditions such as endocarditis, osteomyelitis, pneumonia, brain abscesses, meningitis, and bacteremia [2].

*S. aureus *carries a wealth of pathogenic determinants, which promote tissue colonization, tissue damage, and distant diseases [3–5]. *S. aureus *is able to survive inside host cells and can invade *in vitro* a variety of nonprofessional phagocytes, including fibroblasts [6], osteoblasts [7], endothelial [8], and epithelial cells [9, 10]. After internalization, *S. aureus *may either persist, escaping host defenses and antibacterial agents, or multiply and further disseminate. This behavior is orchestrated by global regulators, which sense environmental modifications, such as bacterial density, and may or may not trigger the secretion of proteins that lyse the host cells and allow the bacteria to propagate [11–14]. Thus, invading host cells might not only provide a therapeutic sanctuary, but also be part of a subtle hide-and-seek strategy, as observed with enteric bacteria [15].

To prevent colonization by inhaled microorganisms, the respiratory epithelium maintains an effective antimicrobial environment by mucociliary clearance and by producing antimicrobial peptides, surfactant proteins, complement, chemokines, and cytokines mediating immune cell recruitment and inflammation [16–18]. All of the innate defense mechanisms of the mammalian airways appear to be directly or indirectly activated by contact of bacterial factors with the epithelial cell surface receptors, which may activate various intracellular signaling pathways. It has long been recognized that *S. aureus* evokes an intense host response dominated by polymorphonuclear leukocytes (PMNs). The induction of genes encoding the proinflammatory cytokines requires activation of mitogen-activated protein kinases (MAPKs) and the transcription factors activator protein-1 (AP-1) and nuclear factor *κ*B (NF-*κ*B) [19–22]. The virulence of *S. aureus* is attributed to many factors. Some of them are implicated in lung infection and have been known for several years. However, the information published in the recent past demonstrated a new pathogenic properties related to known virulence determinants of *S. aureus*. Better understanding of functions and mechanisms of action of each virulence factor is important for improving prognosis of individuals suffering from pneumonia.

In this paper, we summarize recent advance in our understanding of known virulence factors and their role in the initiation of lung inflammation. 

## 2. *S. aureus* Is a Pathogen Implicated in Pneumonia

Over the past 90 years, *S. aureus* has been increasingly recognized as an important cause of pneumonia in both adult and pediatric populations [23–25]. Along with bacteremia,* S. aureus* pneumonia is one of the most prevalent methicillin-resistant *S. aureus*- (MRSA-) related diseases, and the incidence of severe pneumonia caused by MRSA strains rises [26, 27]. Previously, MRSA infections were largely nosocomial infections and a common cause of ventilator-associated pneumonia (VAP), a subtype hospital-acquired pneumonia characterized by high morbidity and mortality [28, 29]. However, in the last few years, there was a dramatic increase in the incidence of community-associated MRSA (CA-MRSA) infections in otherwise healthy individuals and in patients who do not establish risk factors for MRSA, and now, CA-MRSA becomes a common and serious health problem [29]. CA-MRSA strains can cause a necrotizing pneumonia, a specific disease entity that often follows an influenza infection. The necrotizing pneumonia is a rapid progressive form of extensive pneumonia leading to acute respiratory distress with pleural effusion, hemoptysis and leucopenia [24]. Moreover, pneumonia caused by *S. aureus* is a serious complication in individuals with cystic fibrosis and patients affected by immunosuppressive therapy [22, 26, 30, 31]. 

A characteristic manifestation of *S. aureus*-caused pneumonia is the intense host inflammatory response characterized by a rapid and excessive recruitment of neutrophils to the site of infection [32, 33]. In fact, accumulating evidence suggests that disease progression in bacterial pneumonia is largely mediated by the dysregulated and exaggerated host inflammatory response to infection that causes lung injury [34, 35]. Because of the high incidence of pneumonia accompanying with high mortality, it is important to gain more insight into the pathogenesis of this prominent infectious disease.

## 3. Virulence Factors of *S. aureus*

The broad range of infections caused by *S. aureus* is related to a number of virulence factors that allow it to adhere to surface, invade or avoid the immune system, and cause harmful toxic effects to the host [3, 36]. 

### 3.1. Adherence Factors (Adhesins)

The attachment of *S. aureus* to the host cell surface initiating the colonization process is mediated by several adhesins. One major class of *S. aureus* adhesins comprises proteins covalently anchored to cell peptidoglycans (via the threonine residue in the sorting signal motif at their C-terminus), which specifically attach to the plasma or extracellular matrix (ECM) components and collectively are termed the microbial surface component recognizing adhesive matrix molecules (MSCRAMMs) [4, 37–39]. These molecules recognize the most prominent components of the ECM or blood plasma, including fibrinogen, fibronectin, and collagens [3, 40–42].

Typical members of the MSCRAMM family are staphylococcal protein A (SpA), fibronectin-binding proteins A and B (FnbpA and FnbpB), collagen-binding protein, and clumping factor (Clf) A and B proteins [3, 4].

### 3.2. S. aureus Exoproteins

Nearly all strains of *S. aureus* secret a group of exoproteins such as exotoxins and enzymes, including nucleases, proteases, lipases, hyaluronidase, and collagenase. The main function of these proteins may be to convert local host tissue into nutrients required for bacterial growth [5]. 

*S. aureus *produces exotoxins that possess cytolytic activity. Cytolytic toxins form *β*-barrel pores in the plasma membrane and cause leakage of the cell's content and lysis of the target cell [43]. *S. aureus *secrets several cytolytic toxins, among them *α*-hemolysin, *β*-hemolysin, *γ*-hemolysin, leukocidin, and Panton-Valentine leukocidin (PVL) [44]. *α*-hemolysin became inserted into the eukaryotic membrane and oligomerizes into a *β*-barrel that forms a pore which causes osmotic cytolysis and is particularly cytolytic toward human platelets and monocytes [45]. PVL is classified as a bicomponent cytolysin (LukF-PV and LukS-PV) that insert itself into the host's plasma membrane and hetero-oligomerize to form a pore. PVL exhibits a high affinity toward leukocytes, while other bicomponent toxins, *γ*-hemolysin and leukocidin, are cytotoxic toward erythrocytes and leukocytes, respectively [44].

*S. aureus* produces additional group of exotoxins, which include the toxic shock syndrome toxin-1 (TSST-1), the staphylococcal enterotoxins (SEA, SEB, SECn, SED, SEE, SEG, SEH, and SEI) and the exfoliative toxins (ETA and ETB). Among them, TSST-1 and the staphylococcal enterotoxins belong to the group of toxins known as pyrogenic toxin superantigens (PTSAgs) [46, 47]. The best characterized property of this group is superantigenicity, which refers to the ability of this toxin to stimulate proliferation of T-lymphocytes. These toxins cause toxic shock syndrome and food poisoning. ETA and ETB are involved in staphylococcal scalded skin syndrome (SSSS) [48]. The exfoliative toxins have been recognized for long time to possess mitogenic activity toward T lymphocytes [49], but it remains still controversial, whether they should be implicated as superantigens.

*S. aureus* has also other specific proteins that can have profound impact on the innate and adaptive immune system. Examples of such kind of proteins are the staphylococcal complement inhibitor (SCIN), chemotaxis inhibitory protein of *S. aureus* (CHIPS), staphylokinase (SAK), extracellular fibrinogen binding protein (Efb), extracellular adherence protein (Eap), and formyl peptide receptor-like-1 inhibitory protein (FLIPr). SCIN is a C3 convertase inhibitor, which blocks the formation of C3b on the surface of the bacterium and, thereby, the ability of human neutrophils to phagocytose *S. aureus* [50]. CHIPS and FLIPr block neutrophil receptors for chemoattractants [51, 52], Epa blocks migration of neutrophils from blood vessels into the tissue [53], SAK binding to *α*-defensins abolishes their bactericidal properties [54], while Efb inhibits both classical and alternative pathways of complement activation [55, 56].

The virulence of *S. aureus* is generally considered to be multifactorial and due to the combined action of several virulence determinants. One exception is the toxinoses, such as toxin shock syndrome, SSSS, and staphylococcal food poisoning, which are caused by toxic shock syndrome toxin, exfoliative toxins A and B, and different staphylococcal enterotoxins, respectively [3].

In *S. aureus*-induced VAP, multiple virulence factors are implicated. Through the action of LTA, PepG, MSCRAMMs, particularly Fnbp and SpA, and *α*-toxin, *S. aureus* is able to adhere to respiratory epithelium, to damage the alveolocapillary barrier, and to attract PMN [57]. In turn, necrotizing pneumonia is associated with an action of SpA, *α*-toxin, and *β*-toxin, which cause cell damage and play a role in inflammation and necrosis of the respiratory epithelium [32, 35, 58]. The role of PVL in necrotizing pneumonia is controversial.

### 3.3. Regulation of Virulence Factors in S. aureus

The pathogenicity of *S. aureus* is a complex process involving a diverse array of extracellular and cell wall components that are coordinately expressed during different stages of infection (i.e., colonization, avoidance of host defense, growth and cell division, and bacterial spread) [59, 60].

The coordinated expression of diverse virulence factors in response to environmental cues during infections (e.g., expression of adhesins early during colonization versus production of toxins late in infection to facilitate tissue spread) hints at the existence of global regulators in which a single regulatory determinant controls the expression of many unlinked target genes [61]. These regulators help bacteria to adapt to a hostile environment by producing factors enabling the bacteria to survive and subsequently to cause infection at the appropriate time.

Among the environmental signals, changes in nutrient availability, temperature, pH, osmolarity, and oxygen tension have the greatest potential to influence the expression of virulence factors [60]. Production of *S. aureus *virulence determinants is controlled by several global regulatory loci, such as accessory gene regulator (*agr*) [62, 63], staphylococcal accessory regulator (*sarA*) [64, 65], *sae *[66], *sigB* [67, 68], *arl* [69], and number of sarA homologues [70, 71]. These regulators are parts of an important network modulating the expression of *S. aureus *virulence genes. One target virulence gene can be under the influence of several regulators that “cross talk” to ensure that the specific gene is expressed only when conditions are favorable. For instance, *in vitro* studies have demonstrated that *agr *negatively regulates the expression of *spa*, which encodes SpA [71], whereas SarS binds to the *spa *promoter and activates its expression [72]. Interestingly, *agr *downregulates *sarS *expression [65, 72]. Thus, it has been proposed that *agr *downregulates *spa *expression by suppressing the expression of its activator, *sarS *[72]. Therefore, virulence gene regulators could affect the expression of target genes directly, by binding to their promoters, or indirectly, via other regulators.

## 4. Known Virulence Factors of *S. aureus* and Their Novel Functions in Pneumonia

The majority of initial inflammatory responses to inhaled bacteria is signaled by mucosal cells lining the respiratory tract. *S. aureus* has a potential to activate the host inflammatory response in several different ways: through the adherence of intact bacteria to the host epithelial cells, by internalization of the bacteria and by direct interaction of bacterial adhesins and toxins with the mucosal epithelium. The main virulence factors that have potential to cause tissue injury and inflammation in the lung are SpA, *α*-toxin, *β*-toxin, and PVL [24, 32, 73–75]. 

### 4.1. SpA

SpA is a good example of one of known and well-characterized *S. aureus* virulence factors that have recently revealed new properties and play a chief role in the induction of pneumonia. Since many years, SpA is known to be a 42-kDa protein covalently anchored in the bacterial cell wall. It belongs to the MSCRAMM family, because it can bind to the von Willebrand factor, a large glycoprotein that mediates platelet adhesion at sites of endothelial damage [42]. SpA comprises five repeated domains (E, D, A, B, and C), each of them binding with high affinity to the Fc region of immunoglobulin (Ig) G and to the Fab region of Ig of the VH3 subclass [76, 77]. The interaction with Fc of IgG hinders phagocytosis, because bacteria coated with IgG in an inappropriate conformation becomes not recognizable by the Fc receptor on PMN [43]. An additional consequence of the ability of SpA to bind to B lymphocytes displaying IgM bearing VH3 heavy chains is the induction of proliferation resulting in depletion of a significant part of the B cells repertoire [78, 79].

Although the interactions between SpA and Ig chains have long been recognized, only recent studies reveled the central importance of SpA in the pathogenesis of *S. aureus*-induced pneumonia [32, 80, 81]. The absence of SpA reduces pneumonia incidents and associated mortality in a mice model of infection [32].

**Figure 1:**
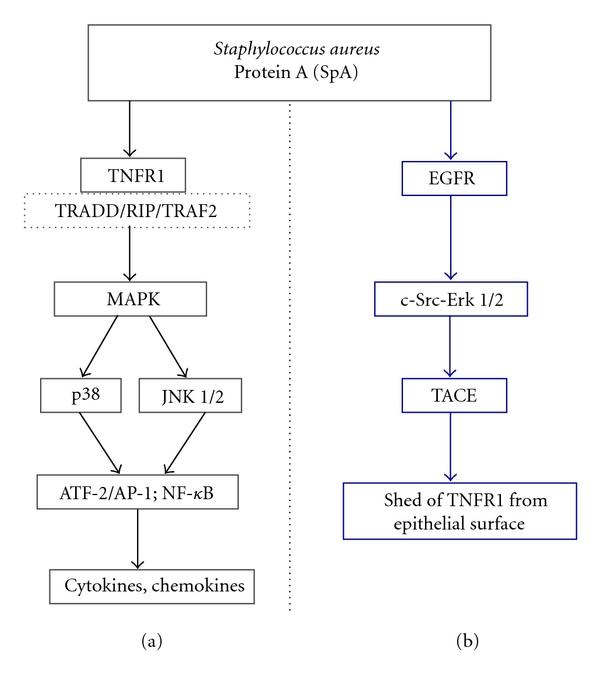
Role of SpA in TNFR1 regulation. (a) SpA is recognized by TNFR1 and the signaling cascade is initiated through the adaptor proteins TRADD/RIP/TRAF2, which subsequently activate MAPK kinases (p38 and JNK 1/2) and induce translocation of transcription factors AP-1 and NF-*κ*B into the nucleus. Activation of AP-1 and NF-*κ*B leads to transcription of genes encoding proinflammatory cytokines and chemokines. (b) SpA through interaction with EGFR and activation of c-Src-Erk1/2 stimulates the activity of TACE (ADAM-17), which cleaves and releases TNFR1 from the airway surface. TNFR1 is then available to neutralize free SpA and TNF-*α* ligands. AP-1, activator protein 1; ATF-2, activating transcription factor 2; EGFR, epidermal growth factor receptor; NF-*κ*B, nuclear factor *κ*B; RIP, receptor-interacting protein; TACE, tumor necrosis factor-*α*-converting enzyme; TNFR1, tumor necrosis factor receptor 1; TRADD, tumor necrosis factor receptor- (TNFR-) associated death domain; TRAF2, tumor necrosis factor receptor-associated factor 2.

Apart of SpA interfering with opsonization by binding to the Fc portion of immunoglobulins, SpA was postulated to have a direct effect on the respiratory epithelial cells even in the absence of IgG. In the infection of the airways where serum components are lacking, SpA plays a chief role in the pneumonia by induction of interleukin- (IL-) 8 expression, and recruitment of PMN into the airway [32]. Although several receptors for SpA, including von Willebrand factor and the platelet protein Gc1qR/p33, have been reported, they, however, are not responsible for the accumulation of PMN in the airways. Tumor necrosis factor- (TNF-) *α* receptor 1 (TNFR1) is widely expressed at the airway epithelium, and its accessibility on the epithelial surface makes it an attractive candidate for mediating host response induced by SpA. An exciting recent study of Gómez et al. [32] showed that SpA interacts directly with TNFR1 and mimics TNF-*α* proinflammatory signaling by recruitment of the adaptor molecules the TNFR-associated death domain (TRADD), receptor-interacting protein (RIP), and TNFR-associated factor (TRAF) 2 to the receptor and the activation of the mitogen-activated protein kinases (MAPKs) p38 and c-Jun NH2-terminal kinases 1 and 2 (JNK1/2), which induces translocation and activation of transcriptional factor NF-*κ*B and mediates IL-8 gene expression. Moreover, SpA-TNFR1 interaction leads to phosphorylation of the activating transcription factor 2 (ATF-2), a component of the AP-1 transcription complex that is regulated through phosphorylation by p38 and JNK1/2 MAPKs (Figure 1(a)). Additionally, TNFR1-deficiency results in reduced morbidity and mortality in a mouse *S. aureus *pneumonia model [32]. Interestingly, in dominant-negative Toll-like receptor (TLR)2 and TLR4 mutants, SpA still induces NF-*κ*B activation in the airway epithelial cells, suggesting that SpA is not TLR2 or TLR4 agonist [32].

#### 4.1.1. Regulation of Inflammation by TNFR1 Shedding

The abundance of TNFR1 is controlled by its mobilization from intracellular stores and cleavage from the cell surface [82–86]. During staphylococcal pneumonia, TNFR1 is specifically mobilized to the apical surface of the airway epithelial cells, providing access to inhaled staphylococci [36]. Cleavage of TNFR1 is known to be mediated by the TNF-*α* converting enzyme (TACE), a central regulator of TNF-*α* signaling [82, 87, 88]. 

TACE (also known as a disintegrin and metalloprotease (ADAM) 17) is a member of the ADAM family of proteases involved in release of several cell surface proteins, including receptors for TNF-*α*, the epidermal growth factor (EGF) and IL-6 [87]. TACE plays an important role in the regulation of inflammation by its ability to cleave and release the extracellular portion of TNFR1 from the surface of airway epithelial cells and macrophages. Shed of TNFR1 from the epithelial surface prevents ongoing signaling and serves to neutralize free TNF-*α* as well as SpA in the airway lumen, and, consequently, the loss of the receptor from the cell surface prevents further epithelial activation.

SpA also induces TACE-dependent cleavage of TNFR1 into the extracellular compartment [32]. Activation of TACE depends on a discrete interaction between SpA and EGF receptor (EGFR), which in turn induces TACE phosphorylation through a c-Src-Erk1/2-mediated cascade (Figure 1(b)) [89]. While TACE is highly expressed on the apical surface of the airway epithelial cells, the substrate, TNFR1, has to be mobilized to the surface, where it colocalizes with TACE. Interaction between EGFR and bacterial SpA and the consequent activation of TACE serve to counteract the proinflammatory consequences of TNFR1 signaling, PMN recruitment and activation. Thus, activation of the TNFR1 pathway not only stimulates mobilization of PMN, but also provides a mechanism to regulate SpA-induced recruitment of neutrophils [32].

Therefore, SpA is involved in the *S. aureus* pneumonia by activating TNFR1 and inducing PMN infiltration that is deleterious to the host. The discovery of the new SpA- TNFR1 signaling axis highlights additional molecular targets to modulate the host immune response and to treat *S. aureus*-caused pneumonia. 

### 4.2. Toxins of S. aureus

*S*.* aureus* *α*-toxin, *β*-toxin, and PVL play an essential role in pneumonia and lung injury. Both, *α*-toxin and PVL, are pore-forming toxins, which exaggerate the host inflammatory response by inducing the expression of proinflammatory cytokines and lysing inflammatory cells to release additional inflammatory mediators. Thus, these toxins have both direct and indirect means to cause a lung damage [73, 90–92]. However, little is known about the significance of these toxins in* S. aureus*-induced pneumonia and lung injury.

#### 4.2.1. *α*-Toxin (*α*-Hemolysin)

*α*-toxin is the major cytotoxic agent released by *S. aureus*, and it was the first bacterial exotoxin to be identified as a pore former [93]. Pore formation on susceptible host cell membranes triggers alterations in ion gradients, loss of membrane integrity, activation of stress-signaling pathways, and cell death [93, 94].

*S. aureus* *α*-toxin is known to play an important role in the pathogenesis of staphylococcal diseases, as *S. aureus* mutants lacking *hla* display reduced virulence in invasive disease models [95]. Interestingly, the dosage of the toxin can result in two different modes of activity. Low concentrations bind to specific cell surface receptors and form a heptameric pore. This pore allows the exchange of monovalent ions, resulting in DNA fragmentation and, eventually, in apoptosis [96]. High concentrations result in the toxin absorbing nonspecifically to the lipid bilayer [97, 98] and forming large, Ca^2+^-permissive pores. This results in massive necrosis and other secondary cellular reactions triggered by the uncontrolled Ca^2+^ influx [96]. 

*α*-toxin is secreted as a water-soluble monomer that undergoes a series of conformational changes to generate a heptameric, *β*-barrel structure in host membranes. Structural maturation of Hla depends on its interaction with a previously unknown proteinaceous receptor. Recently, Wilke and Wanderburg [99] reported that *α*-toxin binding to eukaryotic cell requires ADAM 10 expression to initiate the sequence of events (see below).

*α*-toxin possesses additional biological functions such as binding to a putative glycoprotein receptor on host cells, activation of intracellular signaling, and modulation of several processes [91–93, 96, 100]. It was recently described, that *α*-toxin facilitates the secretion of newly synthesized chemokines into the airway and exaggerates neutrophil-mediated inflammatory lung injury through syndecan-1 ectodomain shedding (see below) [58].

#### 4.2.2. ADAM 10 in S. aureus *α*-Toxin-Mediated Cytotoxicity

Recently, it has been reported that *α*-toxin-ADAM 10 interaction identifies ADAM 10 as the likely proteinaseous cellular receptor for the toxin, which is required for *α*-toxin-mediated cytotoxicity when the toxin is present at low concentrations. Multiple lines of evidence confirm the importance of the membrane lipid environment in *α*-toxin-induced injury, because the membrane opposed region of the toxin interacts with phosphatidylcholine [101], and cholesterol/sphingomyelin-rich membrane domains [102]. It has been shown that clustered phosphocholine head groups serve as the high-affinity binding site for *α*-toxin and provide a mechanistic view of the assembly of *α*-toxin, suggesting that its initial interaction with ADAM10 and the plasma membrane directs the assembly of the *α*-toxin-ADAM10 complex in cholesterol/sphingolipid-rich caveolar rafts. This clustering likely increases the local concentration of *α*-toxin, permitting caveolin 1-directed oligomerization of the toxin and providing accessibility to caveolae-associated proteins FAK and Src, which mediate the biologic effects of *α*-toxin. Focal adhesion disruption by the *α*-toxin-ADAM10 complex provides a mechanism by which the toxin may perturb cellular barriers to cause invasive disease and facilitate superantigen permeation through impenetrable stratified cell layers [103].

#### 4.2.3. *β*-Toxin (*β*-Hemolysin)

Among *S. aureus* toxins, the least is known about the function of *β*-toxin in pneumonia and lung injury. Based on literature data, *S. aureus β*-toxin is a Mg^2+^-dependent neutral sphingomyelinase that hydrolyzes sphingomyelin of the host cell plasma membrane to generate phosphocholine and the bioactive secondary messenger, ceramide [104–106]. Depending on the chain length of their fatty acids or the mode of metabolism, these ceramides may have a number of effects in eukaryotic cells, including stimulation of second messenger systems, activation of MAPKs, changes in cell shape, and even apoptosis [107, 108].

*β*-toxin does not lyse most types of host cells but leaves them susceptible to a number of other lytic agents, such as *α*-toxin and PVL [35]. In fact, the cytotoxic effect of *β*-toxin is cell type-specific and species-specific, suggesting that its primary virulence activity is to modulate host processes that affect pathogenesis, rather than to directly kill host cells [35]. Study of Hayashida et al. [35] uncovered a previously unknown *in vivo* function of *β*-toxin in *S. aureus* pneumonia. *S. aureus* *β*-toxin has been shown to maximize lung injury not through its cytotoxic activity, but rather through its capacity to enhance PMN infiltration in a syndecan-1-dependent manner (see below). Moreover, this toxin can activate different, as yet unknown, cell signaling pathways involved in the induction of c-Fos expression through the NF-*κ*B and p38 MAPK signaling cascades [94, 109–111].

#### 4.2.4. Activation of Syndecan-1 Ectodomain Shedding by S. aureus *α*- and *β*-Toxins

Ectodomain shedding is a proteolytic mechanism of releasing the extracellular domains of cell surface proteins as soluble ectodomains that can regulate many pathophysiological processes, such as microbial pathogenesis, inflammation, and tissue repair [112, 113]. The diverse list of shed proteins includes cytokines, growth factors, and cell adhesion molecules, including TNF-*α*, transforming growth factor-*α* (TGF-*α*), EGF, L-selectin, CD44, and syndecans. *S. aureus* and other bacterial pathogens activate ectodomain shedding of cell surface molecule syndecan-1 to enhance their virulence [35, 58, 100]. Syndecan-1 is the major heparan sulfate proteoglycan of epithelial cells, which binds and regulates a wide variety of biological molecules through its heparan sulfate chains [114]. Both *α*-toxin and *β*-toxin shed syndecan-1 ectodomains through stimulation of the host cells shedding machinery [35, 58, 100]. Several independent lines of evidence suggest that the primary function of syndecan-1 in *α*- and *β*-toxin-induced inflammation is to facilitate PMN infiltration through the generation of chemotactic signals [35, 58].

Forming the small discrete pores by *α*-toxin may trigger syndecan-1 shedding [91, 100]. *α*-toxin does not directly shed syndecan-1 ectodomains, but rather stimulates an endogenous mechanism which involves protein tyrosine kinases (e.g., Syk), but not protein kinase C and MAPK signaling pathways, that enhance the cleavage of syndecan-1 ectodomains by host cell metalloproteinase [100]. Staphylococcal *β*-toxin enhances syndecan-1 shedding by activating ceramide production in the alveolar epithelial cells and by implicating protein tyrosine kinases Syk and JAK2, Erk-type MAPKs, and metalloproteinase [115, 116].

The mechanism of syndecan-1 shedding was well characterized in a mouse model. In bleomycin-induced acute inflammation and lung injury, shedding of syndecan-1 by metalloproteinase-7 generates a chemokine gradient that attracts PMN into the alveolar compartment [117]. Lung injury caused by bleomycin induces the expression of the CXC chemokine KC (CXCL1, mouse functional homologue of human IL-8) and metalloproteinase-7. Newly synthesized KC binds to the heparan sulfate proteoglycans of syndecan-1, and shedding of the syndecan-1/ectodomain-KC complex by metalloproteinase into the alveolar space generates a chemokine gradient across the alveolar epithelial border.

Both *S. aureus* toxins exaggerate lung injury and inflammation through its capacity to enhance neutrophil infiltration [35, 58]. Thus, the shedding of syndecan-1 mediated by *α*- and *β*-toxins may be a critical mechanism in development of a broad range of acute inflammatory disorders.

### 4.3. PVL

Panton-Valentine leukocidin is one of several extracellular cytotoxins produced by *S. aureus*. The toxin was first described by Van de Velde (1894), but only in 1932 Panton and Valentine associated the leukotoxin with skin and soft-tissue infection. Clinical studies propose the exotoxin PVL being a virulence factor in necrotizing diseases [24, 118].

Previous studies revealed that human and rabbit neutrophils are highly sensitive to the pore-forming properties of PVL and rapidly undergo cell death [119]. Furthermore, it is generally accepted that myeloid cells are the prime target of PVL and that low concentrations of the toxin cause apoptosis, whereas higher amounts induce lysis of neutrophils [120]. 

Pore formation requires the presence of the two components of the toxin, LukS-PV and LukF-PV. This pore is an octameric *β*-barrel molecular complex perpendicular to the plane of the cell membrane, similar to that made by *S. aureusα*-toxin [121, 122]. Sublytic concentrations of purified PVL induce pronounced histamine release from human basophils and stimulate human neutrophils to release enzymes (*β*-glucuronidase and lysozyme), chemotactic components (leukotriene-B4 and IL-8), and oxygen metabolites [121, 123, 124]. 

#### 4.3.1. PVL Role in Pneumonia

More than 20 years ago, it was suggested that this lytic toxin functions as a virulence factor in cutaneous infection [125, 126]. Necrotizing pneumonia has long been recognized, but the association with PVL was made by Gillet et al. [24], and numerous cases have been reported worldwide [24, 26, 118, 127–131]. Patients with PVL-positive *S. aureus* in their lungs develop necrotizing pneumonia and have exceedingly high mortality rates, indicating that PVL might be an important virulence factor [24]. However, several studies that used a diversity of animal models have created conflicting results concerning the role of PVL in pneumonia. 

In one study applying a mouse acute pneumonia model, Labandeira-Rey et al. [73] suggested PVL to be a major virulence factor. Using purified toxin or a laboratory strain of *S. aureus* that overexpressed PVL via a plasmid containing *luk-PV* operon, PVL was shown to affect mouse survival in a pneumonia model. The mice showed symptoms of severe illness. It is of interest that when comparing isogenic *S. aureus* strains lysogenized with either wild-type øSLT or mutated øSLT in which the *lukPV* operon was deleted, no difference in mouse survival was found [73], indicating that PVL does not exhibit a lethal effect when expressed from a single transgenic copy. Labandeira-Rey et al. ascribed to PVL a pronounced global gene regulatory effect [73], with the regulatory changes reminiscent of disrupting the accessory gene regulator *agr* [132]. They showed that the expression of PVL induces global changes in transcriptional levels of genes encoding secreted and cell-wall-anchored staphylococcal proteins, including SpA [73]. It should be mentioned that this statement is controversial: Diep and Otto [133] explained that misinterpretation of the data due to the apparent lack of confirmatory experiments might have led to the model in which PVL plays a role in global gene regulation. Also, other groups fail to detect any pathogenic function of PVL in murine model of pneumonia. Using isogenic Δ*pvl* mutants in the MW2 and USA300 backgrounds, and when overexpressing PVL in *S. aureus* strain Newman, no significant contribution of PVL to lethal pneumonia was found using mice [75, 134]. Moreover, it was suggested that Hla, but not PVL, was essential for the pathogenesis of staphylococcal pneumonia [75]. Passive immunization with anti-PVL immune sera also failed to protect mice against challenge with USA300 in the murine pneumonia model [95], indicating that PVL is not necessary for the pathogenesis of pulmonary disease. 

#### 4.3.2. Role of TLR in PVL-Mediated Lung Inflammation

Despite the role of PVL as a virulence factor in the lungs is controversial, the pulmonary immune response to PVL, especially responsiveness of alveolar macrophages to this toxin, is known [135]. The recent study of Zivkovic et al. [135] showed that PVL induced a highly specific inflammatory transcriptional response in alveolar macrophages. The alveolar macrophages are considered to represent the first line of defense against pathogens and express receptors, including TLRs, which recognize pathogen-associated molecular patterns [136]. Activation of TLRs triggers the MAPK and NF-*κ*B signaling pathways. These pathways further modulate proinflammatory gene expression, which is crucial in shaping the innate immune response within the respiratory tract [137]. The idea that TLRs could play an important role in bacterial toxin recognition is not uncommon. Other pore-forming toxins have been shown to mediate inflammation via TLRs, particularly via TLR2 and TLR4 [138, 139]. Zivkovic et al. [135] demonstrated that PVL directly binds to the extracellular domain of TLR2 and induces immune response via NF-*κ*B in a TLR2, CD14, MyD88, IL-1 receptor-associated kinase 1, and TRAF6-dependent manner. However, in contrast to data showing that LukF from *S. aureus* is able to induce inflammation in a TLR4-dependent manner in bone marrow-derived dendritic cells [140], the study of Zivkovic et al. [135] demonstrated that the active component of the toxin is LukS, because the stimulation of macrophages with LukS, but not with LukF, resulted in an inflammatory response* in vitro *and *in vivo*. Furthermore, overexpression of TLR2, but not CD14, is sufficient for LukS to induce an inflammatory response, indicating that CD14 can act only as a coreceptor.

The ability of PVL to induce inflammatory gene expression is independent of pore formation [135]. These data are in line with previous observations, showing that both subunits of PVL are required to perform a pore [122]. Interestingly, although single subunits are incapable of forming the pore, LukS is capable of inducing TNF-*α* gene expression. Furthermore, single submit LukS, but not LukF, is able to induce an inflammatory response, suggesting that inflammatory gene expression relies on cellular pathways independent of pore formation [135]. 

## 5. Eradication of Infection of *S. aureus* in the Lungs

*S. aureus *deploys a combination of virulence factors, including adhesins, toxins, and immunomodulatory molecules, that facilitate infection of different host tissues [141, 142]. The knowledge about host factors, which facilitate eradication of *S. aureus *in the lungs, is limited.

Surfactant protein A (SP-A) is the major protein component of pulmonary surfactant. It is involved in organization of large aggregates of surfactant phospholipids lining the alveolar surface and acts as an opsonin for pathogens [143]. Previous studies established that SP-A modulates macrophage phagocytosis and a host pro- and anti-inflammatory responses that help in eradication of infection [144–148]. Recent study of Sever-Chroneos et al. [149] demonstrated the role of SP-A in opsonization and clearance of *S. aureus*. Macrophage receptor SP-R210 is implicated in the ability of SP-A to coordinate the clearance of pathogens and apoptotic cells, and to participate in temporal control of inflammation in the lungs [145]. SP-R210 mediates also binding of SP-A-opsonized *S. aureus *by macrophages [149]. Phagocytosis of SP-A-opsonized *S. aureus *via SP-R210 is coordinated with secretion of TNF-*α* and suppression of bacterial growth in macrophages. Furthermore, expression of the staphylococcal adhesin Eap is necessary for both SP-A binding and enhanced phagocytosis of SP-A-opsonized bacteria by SP-R210. Finally, Sever-Chroneos et al. [149] revealed previously unknown link between expression of SP-R210 isoforms and the scavenger receptor SR-A. Binding of SP-A to SP-R210S induces phagocytosis and release of anti-inflammatory mediators via association with SR-A, leading to an enhanced bacterial killing and resolution of the infection. 

Based on previous findings, SP-R210 [150] and SR-A [151] may coordinate secretion of IL-10, TGF-*β*, and hydrogen peroxide in alveolar macrophages. Importantly, it is proposed that temporal control of inflammatory responses via SP-R210S and SR-A contributes to the proper recruitment and activation of neutrophils, facilitating eradication of *S. aureus *infection in the lungs. However, moderate levels of hydrogen peroxide may suppress inflammation through inactivation of NF-*κ*B [152, 153] and enhance bacterial killing through activation of NADPH oxidase [154] during the resolution phase of the disease.

## 6. Conclusion

The innate defense of the airway epithelial cells against *S. aureus* includes a regulated secretion of cytokines and chemokines, and involves different signalling pathways. Induction of the airway inflammation can be mediated by several staphylococcal determinants and corresponding receptors and is not necessarily dependent on the expression of a particular virulence factor that is crucial for the pathogenesis of *S. aureus* infection in other body sites.

Among many virulence factors produced by *S. aureus*, SpA, *α*-, and *β*-toxins play an important role in the of pathogenesis of staphylococcal pneumonia. The role of PVL in lung infection is debated due to conflicting data. 

The shedding of the plasma membrane proteins represents an important mechanism underlying *S. aureus* properties in the lungs. *α*-toxin and *β*-toxin of *S. aureus* activate ectodomain shedding of host components to promote bacterial pathogenesis. In addition, the airway epithelial cells regulate their own signaling capabilities by shedding some epithelial receptors (e.g., TNFR1) that serves to bind and neutralize inflammatory cytokines released by immune cells.

Considerable progress has been made in our understanding of known virulence factors and their implication in pneumonia in the last few years. Several new properties of *S. aureus* virulence determinants have been identified. A detailed analysis of function and mechanisms of action of each virulence factor could open the way to control the proinflammatory response in the lung by using specific inhibitors and may be helpful for the development of novel therapies for *S. aureus*-caused pulmonary diseases.

## Abbreviations:

CHIPS: Chemotaxis inhibitory protein of *S. aureus *
Clf A, B: Clumping factor A and B
Eap: Extracellular adherence protein
Efb: Extracellular fibrinogen-binding protein
ET A, B: Exfoliative toxins A and B
FLIPr: Formyl peptide receptor-like-1 inhibitory protein
IL: Interleukin
EGF: Epidermal growth factor
EGFR: Epidermal growth factor receptor
MAPKs: Mitogen activated protein kinases
MSCRAMMs: Microbial surface component recognizing adhesive matrix molecules
PVL: Panton-Valentine leukocidin
PTSAgs: Pyrogenic toxin superantigens
RIP: Receptor-interacting protein
SAK: Staphylocinase
SCIN: Staphylococcal complement inhibitor
SpA: Staphylococcal protein A
SP-A: Surfactant protein A
TACE: Tumor necrosis factor-converting enzyme
TLR: Toll-like receptor
TNF-*α*: Tumor necrosis factor alpha
TNFR1: Tumor necrosis factor alpha receptor 1
TRADD: Tumor necrosis factor alpha receptor-associated death domain
TRAF: Tumor necrosis factor alpha receptor-associated factor
TSST-1: Toxic shock syndrome toxin-1.
